# Functional Ureteral Obstruction Due to Retroperitoneal Tissue Interposition During Oblique Lumbar Interbody Fusion: A Report of Two Cases

**DOI:** 10.3390/jcm15093235

**Published:** 2026-04-23

**Authors:** Jun-Seok Lee, Young-Hoon Kim, Sang-Il Kim, Kihyun Kwon, Sangjun Park, Joonghyun Ahn, Chungwon Bang, Hyung-Youl Park

**Affiliations:** 1 Department of Orthopedic Surgery, Eunpyeong St. Mary’s Hospital, College of Medicine, The Catholic University of Korea, Seoul 03312, Republic of Korea; junband@naver.com; 2 Department of Orthopedic Surgery, Seoul St. Mary’s Hospital, College of Medicine, The Catholic University of Korea, Seoul 06591, Republic of Korea; 3 Department of Orthopedic Surgery, Bucheon St. Mary’s Hospital, College of Medicine, The Catholic University of Korea, Bucheon 14647, Republic of Korea; ajhssnim@gmail.com; 4 Department of Orthopedic Surgery, Incheon St. Mary’s Hospital, College of Medicine, The Catholic University of Korea, Incheon 21431, Republic of Korea

**Keywords:** oblique lumbar interbody fusion, functional ureteral obstruction, retroperitoneal tissue interposition, interbody cage positioning, surgical complication

## Abstract

**Background/Objectives**: Ureteral complications following oblique lumbar interbody fusion (OLIF) are uncommon and are typically attributed to direct mechanical injury. Functional ureteral obstruction without overt ureteral damage remains poorly characterized. We report two cases that provide clinical and intraoperative evidence of a previously underrecognized mechanism of ureteral obstruction associated with anterior cage positioning during OLIF. **Case Presentation**: Among 180 OLIF procedures performed by a single surgeon, two cases (1.1%) of postoperative or intraoperative ureteral compromise without direct structural injury were identified. In the first case, postoperative imaging revealed hydronephrosis and focal angulation of the left proximal ureter at the level of the interbody cage, without contrast extravasation. The obstruction was managed with double-J ureteral stenting, and serial renal function monitoring confirmed preserved renal function throughout the clinical course. In the second case, retroperitoneal tissue including the ureter was directly observed intraoperatively to be interposed between the anterior longitudinal ligament and the interbody cage during anterior cage placement. Release of the interposed tissue resulted in immediate ureteral decompression without structural damage. Correlation of the postoperative findings in the first case with the intraoperative observations of the second case supports a unified mechanistic explanation: anterior cage advancement may draw retroperitoneal tissue into the cage–anterior longitudinal ligament interface, subjecting the ureter to focal compression or angulation. **Conclusions**: Functional ureteral obstruction during OLIF may occur secondary to retroperitoneal tissue interposition rather than direct ureteral trauma. Awareness of this mechanism and meticulous protection of the anterior retroperitoneal layer during cage advancement may help prevent avoidable ureteral complications.

## 1. Introduction

Oblique lumbar interbody fusion (OLIF) has become a widely adopted minimally invasive technique for the treatment of degenerative lumbar spine disorders [1]. By accessing the disc space through the anatomical corridor between the psoas muscle and the major abdominal vessels, OLIF allows for the insertion of large interbody cages, thereby restoring disc height, correcting segmental alignment, and achieving effective indirect decompression while minimizing posterior muscle disruption [2,3]. Owing to these advantages, OLIF has gained increasing popularity in the surgical management of various lumbar pathologies [4].

Although neurological complications related to the psoas muscle and lumbar plexus have been well documented, non-neurological complications involving adjacent retroperitoneal structures are relatively uncommon but clinically significant [5,6,7]. Among these, iatrogenic ureteral injury represents a potentially serious complication that may result in delayed diagnosis and substantial morbidity [8]. Previously described ureteral complications during OLIF have primarily involved direct mechanical trauma, such as transection, ligation, or thermal injury [9,10]. These injuries are generally identifiable intraoperatively or detected early in the postoperative course.

In contrast, ureteral obstruction occurring without overt structural damage is rarely reported and remains poorly understood. While postoperative ureteric entrapment associated with interbody cage placement has been described in isolated cases [7,11], the precise intraoperative mechanism leading to functional obstruction has not been clearly defined. Anatomical studies have demonstrated that the ureter is typically positioned anterior to the psoas muscle in the lateral decubitus position, placing it in close proximity to the anterior boundary of the OLIF surgical corridor [12,13], yet the potential role of retroperitoneal tissue interposition during anterior cage positioning has not been sufficiently emphasized in the literature. The expanding indications and increasing adoption of OLIF have brought renewed attention to the full spectrum of approach-related complications beyond neurological sequelae [14].

We present two cases in which correlation of postoperative imaging findings with direct intraoperative visualization provides insight into this mechanism and suggests technical considerations related to anterior cage positioning and meticulous soft tissue management that may help prevent preventable ureteral complications.

## 2. Case Presentation

### 2.1. Case 1: Postoperative Functional Ureteral Obstruction

This report is based on a retrospective review of two cases of ureteral compromise without direct structural injury identified among 180 OLIF procedures performed by a single surgeon at a single academic institution between January 2020 and December 2025. Postoperative imaging evaluation included MRI, contrast-enhanced CT, and IVP where clinically indicated, and renal function was monitored serially using serum creatinine and blood urea nitrogen levels.

A 64-year-old woman presented with bilateral radiating leg pain that had persisted despite several months of conservative treatment. Preoperative radiographs and MRI demonstrated grade II degenerative spondylolisthesis of L4 on L5, accompanied by lateral recess stenosis at the L4–5 level. Based on the persistence of symptoms and radiologic findings, surgical intervention was indicated.

At the L4–5 level, the retroperitoneal corridor was established through a mini-open approach [15]. The patient underwent OLIF at L4–5 followed by percutaneous posterior pedicle screw fixation. Insertion of the interbody cage restored disc height and achieved partial reduction in the spondylolisthesis, which was further corrected and stabilized following posterior percutaneous pedicle screw fixation. No intraoperative complications, abnormal tissue entrapment, or active bleeding were observed.

**Figure 1:**
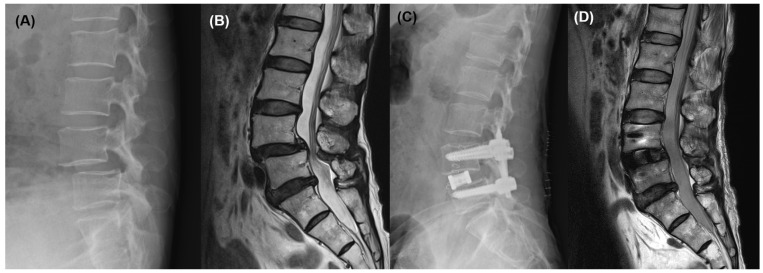
(**A**) Preoperative lateral radiograph demonstrating grade II degenerative spondylolisthesis at L4–5. (**B**) Preoperative sagittal MRI showing lateral recess stenosis at L4–5. (**C**) Postoperative lateral radiograph demonstrating restoration of disc height and reduction in spondylolisthesis after OLIF and posterior percutaneous pedicle screw fixation. (**D**) Postoperative sagittal MRI obtained 12 days after surgery, showing adequate indirect decompression at L4–5.

Postoperative lumbar spine MRI obtained 12 days after surgery demonstrated satisfactory indirect decompression at L4–5 (Figure 1). However, incidental dilatation of the left renal pelvis and proximal ureter was identified. To further evaluate this finding, contrast-enhanced CT was performed, which revealed abrupt luminal narrowing and focal angulation of the left proximal ureter at the L4 vertebral level, adjacent to the anteriorly positioned cage. Associated hydronephrosis was present, but no contrast extravasation or radiologic evidence of ureteral transection was observed, suggesting functional ureteral obstruction rather than direct ureteral injury.

**Figure 2:**
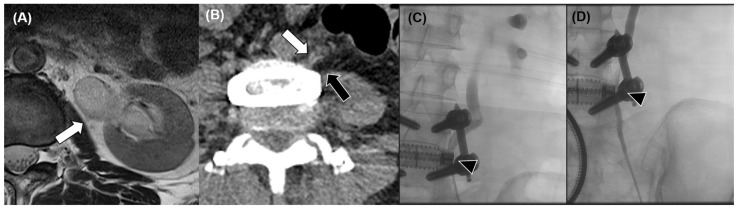
(**A**) Postoperative lumbar MRI demonstrating incidental dilatation of the left renal pelvis and proximal ureter (white arrow). (**B**) Contrast-enhanced CT showing abrupt angulation of the left proximal ureter toward the interbody cage at the L4 level (white arrow), adjacent to retroperitoneal soft tissue (black arrow). (**C**) Intravenous pyelography demonstrating focal narrowing of the ureter at the level of the interbody cage (black arrowhead). (**D**) Follow-up imaging showing improved ureteral narrowing (black arrowhead) with mild residual stenosis.

The patient was managed in collaboration with the urology department. Intravenous pyelography demonstrated no evidence of ureteral disruption. During double-J ureteral stent insertion, significant angulation of the ureter was noted at the level corresponding to the cage insertion, consistent with mechanical kinking. The stent was successfully advanced and maintained for approximately one year. Serial renal function monitoring demonstrated that serum creatinine remained within normal limits throughout the clinical course (preoperative: 0.61 mg/dL; peak postoperative: 0.66 mg/dL; last follow-up: 0.53 mg/dL), confirming the absence of permanent renal impairment despite prolonged ureteral obstruction. Serial follow-up imaging demonstrated gradual resolution of the ureteral narrowing and improvement of hydronephrosis. The stent was eventually removed without recurrence of urinary complications (Figure 2). At the time of initial management, the precise etiology of the ureteral obstruction remained unclear, as no direct structural injury had been identified intraoperatively. The underlying mechanism was subsequently re-interpreted in light of the intraoperative findings observed in Case 2.

### 2.2. Case 2: Intraoperative Evidence of Retroperitoneal Tissue Interposition

A 69-year-old woman with diabetes mellitus and hypertension underwent OLIF for two-level degenerative spondylolisthesis at L3–4 and L4–5. Intraoperative findings in this case provided direct insight into a potential mechanism of ureteral compromise. The L4–5 level was approached using a standard mini-open retroperitoneal technique. Following discectomy and meticulous endplate preparation, a trial cage was inserted to determine the appropriate implant size and positioning. During final cage insertion, the interbody device was advanced toward the anterior portion of the disc space to optimize anterior column support and segmental alignment [16].

**Figure 3:**
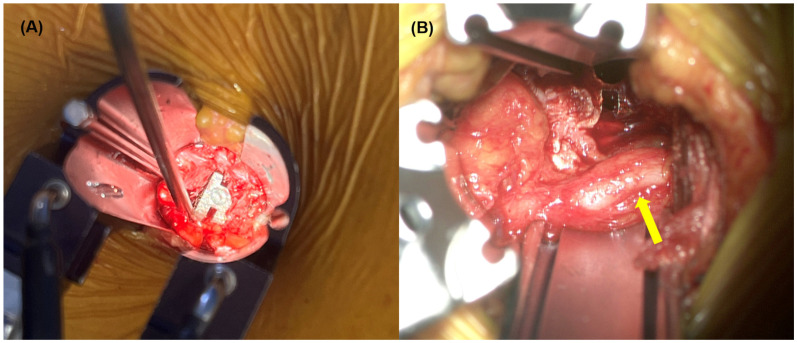
(**A**) Intraoperative photograph showing retroperitoneal tissue interposed between the anterior longitudinal ligament and the interbody cage. The suction tip indicates the interposed tissue. (**B**) After partial release of the anterior longitudinal ligament, the interposed tissue is released, revealing a swollen ureter (yellow arrow).

Immediately after cage placement, retroperitoneal soft tissue was observed to be tightly interposed between the anterior surface of the cage and the anterior longitudinal ligament (ALL) (Figure 3A). The interposed tissue appeared under marked tension. Careful dissection and partial release of the ALL were performed to mobilize the entrapped layer. Upon release, the retroperitoneal tissue was reduced anteriorly, and the ureter was identified within this tissue plane. The ureter demonstrated focal swelling at the precise site of compression (Figure 3B), corresponding to the region where the interposed tissue had been tensioned between the cage and anterior spinal structures. Notably, there was no evidence of ureteral laceration, urine leakage, thermal injury, or disruption of ureteral continuity. These findings indicated transient mechanical compression rather than structural ureteral damage.

After complete release of the entrapped retroperitoneal tissue and confirmation that no residual soft tissue remained interposed between the cage and anterior structures, the procedure proceeded without further difficulty. The remaining surgical steps, including OLIF at L3–4 and posterior percutaneous pedicle screw fixation, were completed uneventfully. Postoperative imaging demonstrated appropriate cage positioning without hydronephrosis or signs of ureteral obstruction.

## 3. Discussion

### 3.1. Proposed Mechanism of Functional Ureteral Obstruction

Among 180 OLIF procedures (278 surgical levels) performed during the study period, two cases of functional ureteral obstruction without direct structural injury were identified (1.1% per patient; 0.72% per level). Both cases involved the L4–5 segment, which was the most frequently operated level in our series (141 of 180 cases, 78.3%), and represented an incidence of 1.4% among patients undergoing OLIF at L4–5.

**Figure 4:**
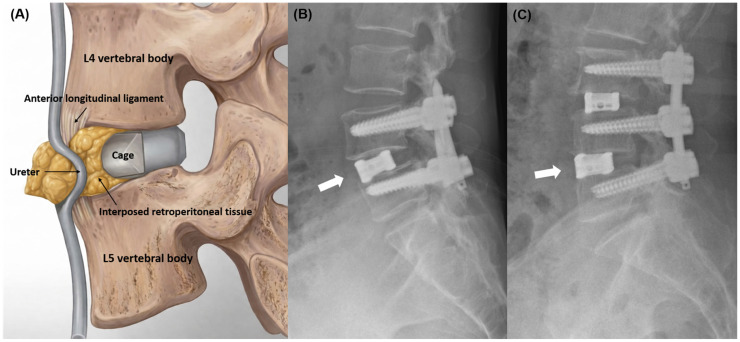
Proposed mechanism of functional ureteral obstruction and anterior cage positioning: (**A**) Schematic illustration demonstrating retroperitoneal tissue interposition between the interbody cage and the anterior longitudinal ligament, subjecting the ureter to focal compression. (**B**,**C**) Postoperative lateral radiographs of Case 1 and Case 2, respectively, demonstrating anterior positioning of the interbody cage at the L4–5 level in both cases. White arrows indicate the anterior margin of the interbody cage approximating the anterior vertebral margin.

The intraoperative observations in Case 2, interpreted in conjunction with the postoperative imaging and renal function data in Case 1, offer a plausible mechanistic explanation for functional ureteral obstruction occurring without direct structural injury (Figure 4A). Anterior advancement of the interbody cage, particularly when performed to optimize anterior column support and sagittal alignment, may alter the spatial relationship between the cage, the ALL, and adjacent retroperitoneal structures [17]. As the cage is advanced toward the anterior margin of the disc space, retroperitoneal soft tissue located ventral to the ALL may be drawn posteriorly and become interposed between the cage and the ligament. If this interposed tissue layer remains under tension, the ureter embedded within the retroperitoneal plane may be subjected to focal compression or acute angulation, resulting in functional obstruction rather than overt injury. Because the ureter remains anatomically intact, intraoperative recognition may be challenging, and postoperative imaging may reveal hydronephrosis or abrupt ureteral angulation without contrast extravasation.

### 3.2. Ureteral Complications in OLIF: Beyond Direct Injury

Despite its widespread adoption as an effective minimally invasive technique for degenerative lumbar disorders [18], OLIF carries a risk of non-neurological complications involving adjacent retroperitoneal structures that remain uncommon and are less frequently recognized [19]. Ureteral complications following OLIF are rare but potentially serious. Previously reported cases have mainly involved direct ureteral injury, including transection or thermal damage [9,10]. However, anatomical and radiological studies suggest that the overall risk of direct ureteral injury during OLIF is relatively low when proper surgical planes are respected. Hamanaka et al. [12] demonstrated that in the lateral decubitus position, the ureter typically migrates anteromedially, resulting in minimal overlap with the conventional OLIF corridor.

Nevertheless, postoperative ureteric obstruction without overt ureteral damage has also been described [7,11]. Wadhwa et al. [11] demonstrated that abrupt ureteral cutoff at the level of the interbody cage ultimately required ureteroureterostomy due to ureteral entrapment. Similarly, Yoon et al. [7] reported a case of delayed ureteral stricture and ipsilateral kidney atrophy detected three months after OLIF, despite the absence of intraoperative injury. However, neither report provided direct intraoperative evidence clarifying the mechanical sequence leading to ureteral compromise. The present cases are, to our knowledge, the first to provide direct intraoperative visual evidence of retroperitoneal tissue interposition as a mechanism of functional ureteral obstruction during OLIF, and to correlate these findings with postoperative imaging and renal function data.

### 3.3. Anatomical Considerations and Technical Implications

The anterior margin of the disc space lies in close anatomical proximity to the retroperitoneal space [20]. Fujibayashi et al. [13] demonstrated that the ureter is typically positioned anterior to the psoas muscle, placing it near the anterior boundary of the surgical corridor. In the lateral decubitus position required for OLIF, the ureter migrates anteromedially relative to its supine position, theoretically reducing—but not eliminating—the risk of ureteral contact with the surgical field [12]. When the interbody cage is advanced toward the anterior disc margin, this spatial relationship becomes particularly relevant, as retroperitoneal tissue may be drawn into the cage–ALL interface. In both cases presented herein, postoperative lateral radiographs demonstrated that the interbody cage was positioned with its anterior margin approximating the anterior vertebral margin at the L4–5 level (Figure 4B,C), consistent with anterior zone placement as defined by Park et al. [16] and Qiao et al. [17]. This degree of anterior positioning is considered a predisposing factor for retroperitoneal tissue interposition, as it places the cage in closest proximity to the ALL and the adjacent retroperitoneal structures.

From a technical perspective, careful inspection of the anterior disc space and the retroperitoneal layer is essential both immediately before cage advancement and after final cage seating. After completing endplate preparation, we recommend performing a deliberate “anterior clearance check” by gently sweeping the retroperitoneal tissue away from the anterior disc margin and confirming that the ALL is clearly visualized without soft tissue interposition. During cage advancement, a dedicated anterior retractor (e.g., a nerve root retractor or a narrow-blade retractor) can be placed along the anterior aspect of the ALL to act as a physical barrier, maintaining a protected plane and preventing retroperitoneal tissue from being drawn into the cage–ALL interface. This maneuver is particularly relevant when the cage is intentionally advanced anteriorly for alignment goals [16,17]. If tight soft tissue interposition is identified, the priority should be to avoid forceful advancement or repeated impaction, which may exacerbate entrapment. Instead, stepwise reduction can be achieved by slightly backing off the cage, re-establishing anterior clearance, and then re-advancing under direct visualization. When interposed tissue remains tensioned, cautious and limited release of the ALL may facilitate safe reduction in the entrapped layer while minimizing secondary injury [21]. In selected cases where anterior cage positioning is planned, controlled ALL release or anterior soft tissue mobilization before final cage advancement may serve as a preventive strategy [22].

**Figure 5:**
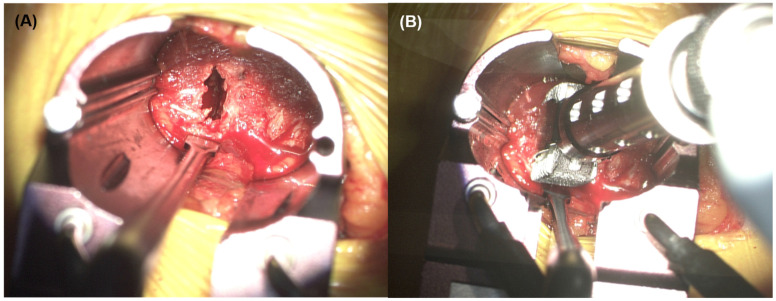
(**A**) After discectomy, a retractor is placed along the anterior aspect of the anterior longitudinal ligament to prevent interposition of retroperitoneal fat into the disc space. (**B**) Interbody cage insertion is performed with the retractor maintained in position, providing anterior soft tissue protection during cage advancement.

Since recognizing this potential mechanism, we have modified our surgical technique by routinely protecting the anterior aspect of the ALL during cage insertion, maintaining continuous anterior soft tissue protection until the cage is fully seated (Figure 5). These precautions are presented as experience-based technical suggestions rather than evidence-based guidelines, given the limited number of cases on which they are founded. However, their rationale is supported by the anatomical and intraoperative observations described herein. Recent advances in single-position OLIF techniques and navigation-assisted cage placement have enabled more precise control of anterior cage positioning, potentially reducing unintended soft tissue interposition [23]. Awareness of this potential mechanism and adoption of protective technical measures may help reduce preventable ureteral complications as OLIF continues to evolve.

### 3.4. Implant Characteristics and Anterior Cage Positioning

**Figure 6:**
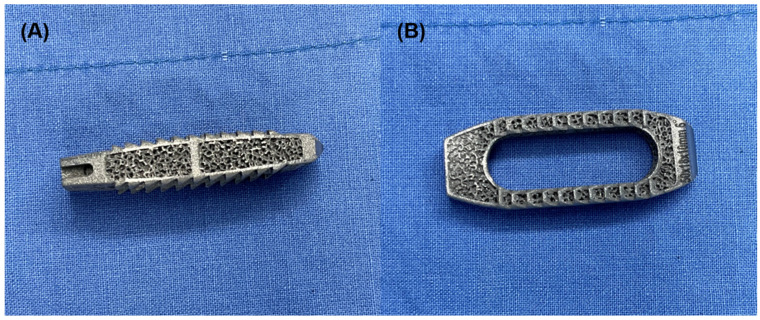
Three-dimensional (3D)-printed titanium interbody cage used in the present cases. (**A**) Anterior (frontal) view of the 3D-printed titanium interbody cage demonstrating the porous lattice architecture and rigid metallic frame design. (**B**) Superior view illustrating the surface topology and structural configuration of the cage.

Although the proposed mechanism is not limited to a specific implant type, it is noteworthy that 3D-printed titanium cages were used in both observations (Figure 6). The rigidity of metallic cages, combined with the surface roughness inherent to 3D-printed structures, may theoretically increase friction during insertion and facilitate mechanical interaction with adjacent soft tissues [24,25]. Such characteristics could potentially enhance the risk of soft tissue interposition when the cage is advanced anteriorly.

However, anterior cage positioning itself likely represents the more critical predisposing factor, regardless of cage material. Notably, review of the radiographic images in previously reported cases of ureteral compromise following OLIF appears consistent with the use of conventional PEEK cages, indicating that this complication is not restricted to metallic or 3D-printed implants [7,11]. This observation further supports the notion that anterior cage positioning, rather than implant surface characteristics, represents the primary predisposing factor. Further investigation would be required to determine whether implant design or surface characteristics independently contribute to the risk of retroperitoneal tissue interposition [26].

### 3.5. Limitations

The present report has several limitations. First, the observations are based on two cases (1.1%) identified from a series of 180 OLIF procedures performed over six years, and do not allow estimation of the true incidence of ureteral compromise associated with anterior cage positioning. As such, the proposed mechanism should be interpreted as hypothesis-generating rather than definitive, and the technical suggestions offered herein reflect the authors’ surgical experience rather than validated preventive protocols. Second, because this report is based on intraoperative observation and postoperative imaging correlation, quantitative assessment of the degree of ureteral angulation or compression was not performed. The absence of direct measurement limits objective validation of the proposed mechanical pathway. Third, although 3D-printed titanium cages were used in both cases, the present report does not establish a causal relationship between implant material and retroperitoneal tissue interposition. The role of implant design and surface characteristics in soft tissue interaction requires further investigation.

Despite these limitations, the intraoperative visualization of retroperitoneal tissue interposition in conjunction with postoperative functional obstruction—and the correlation of these findings with serial renal function data confirming transient and reversible obstruction— provides clinically relevant insight into a potentially underrecognized mechanism of ureteral compromise during OLIF.

## 4. Conclusions

Functional ureteral obstruction may occur following OLIF in the absence of overt structural injury and can be related to retroperitoneal tissue interposition during anterior cage positioning. The present two cases offer intraoperative and radiologic correlation supporting a mechanical mechanism of ureteral angulation or compression rather than direct trauma. Renal function monitoring confirmed that the obstruction was transient and reversible, with no permanent renal impairment. Meticulous inspection and protection of the anterior retroperitoneal layer during cage advancement may help reduce avoidable ureteral complications. Given the limitations inherent to a small case report, these findings are best regarded as hypothesis-generating observations that may inform future prospective studies on the incidence and prevention of this complication.

## Data Availability

The datasets used and/or analyzed during the current study are available from the corresponding author upon reasonable request.

## Abbreviations

The following abbreviations are used in this manuscript:

OLIF	Oblique lumbar interbody fusion
MRI	Magnetic resonance imaging
CT	Computed tomography
ALL	Anterior longitudinal ligament
